# Altering the availability of healthier vs. less healthy items in UK hospital vending machines: a multiple treatment reversal design

**DOI:** 10.1186/s12966-019-0883-5

**Published:** 2019-11-27

**Authors:** Rachel Pechey, Holly Jenkins, Emma Cartwright, Theresa M. Marteau

**Affiliations:** 1 Behaviour and Health Research Unit, Institute of Public Health, University of Cambridge, Cambridge, UK; 2 Oxford University Hospitals NHS Foundation Trust, The John Radcliffe Hospital, Oxford, UK; 3 Nuffield Department of Primary Care Health Sciences, University of Oxford, Oxford, UK

**Keywords:** Absolute and relative availability, Healthiness, Food, Energy, Purchases, Vending machines

## Abstract

**Background:**

Altering the availability of healthier or less-healthy products may increase healthier purchases, but evidence is currently limited. The current study aimed to investigate the impact of altering the absolute-and-relative availability of healthier and less-healthy products – i.e. simultaneously altering the number of options available and the proportion of healthier options – in hospital vending machines.

**Methods:**

An adapted multiple treatment reversal design was used, altering products available in ten vending machines serving snack foods and/or cold drinks in one English hospital. Machines were randomised to one of two sequences for the seven 4-week study periods: ABCADEA or ADEABCA. In Condition A (study periods 1, 4 and 7) the proportions of healthier products were standardised across all machines, so that 25% of all snack slots and 75% of drink slots were healthier. In Condition B, 20% of vending machine slots were emptied by removing less-healthy products. In Condition C, the empty slots created in Condition B were filled with healthier products. Conditions D and E were operationalised in the same way as B and C, except healthier products were removed in D, and then less-healthy products added in E. Sales data were obtained from machine restocking records. Separate linear mixed models were conducted to examine the impact of altering availability on energy purchased (kcal) from (i) snacks or (ii) drinks each week, with random effects for vending machine.

**Results:**

The energy purchased from drinks was reduced when the number of slots containing less-healthy drinks was decreased, compared to standardised levels (− 52.6%; 95%CI: − 69.3,-26.9). Findings were inconclusive for energy purchased from snacks when less-healthy snack slots were reduced (− 17.2%; 95%CI: − 47.4,30.5). Results for altering the number of slots for healthier drinks or snacks were similarly inconclusive, with no statistically significant impact on energy purchased.

**Conclusions:**

Reducing the availability of less-healthy drinks could reduce the energy purchased from drinks in vending machines. Further studies are needed to establish whether any effects might be smaller for snacks, or found with higher baseline proportions of healthier options.

## Background

Overconsumption of food and drink is a key contributor to the high rates of overweight and obesity in the population [1]. One possible intervention for reducing this is altering the availability of foods and drinks by increasing the range of healthier food/drink options and/or decreasing the range of less healthy options. Indeed, availability is one of the top three interventions suggested in the McKinsey Global Institute report [2] as having the highest likely impact across the population, and a Cochrane review of the impact of availability interventions suggests that such interventions can reduce selection and consumption of targeted food products – albeit limited by the quality and quantity of the included studies [3]. Availability can be conceptualised in a number of ways, with interventions altering: (i) Absolute Availability i.e. the overall number of options; (ii) Relative Availability i.e. the proportion comprised by a subset of products; or (iii) Absolute and Relative Availability i.e. both (i) and (ii) above, simultaneously [4].

A recent review of interventions in vending machines found that sales of healthier items were increased in five of the six identified studies that intervened to increase their availability, with no loss of overall sales volume [5]. Similarly, a report by Public Health England suggested that introducing healthier products in hospital vending machines might reduce energy purchased from drinks and snacks – although the study also found increased sugar was purchased from snacks [6]. However, this study was limited by its design and analysis: the positioning of products – healthier and less healthy – was changed simultaneously, and no statistical evaluation was reported. A review of worksite interventions [7] identified only two studies focused on increasing the availability of healthier foods as a single intervention, both showing introducing fruit baskets led to increased fruit intake [8, 9].

As such, while there is some evidence to suggest that increasing the availability of healthier over less healthy foods has the potential to increase purchases of healthier food and drink, more studies are needed to explore this effect further. In particular, studies are needed that are designed to disentangle the effects of availability from other factors, such as price, labelling or positioning, that have often been altered simultaneously [7].

The National Health Service (NHS), one of Europe’s largest employers, has committed to improving the health of its workforce [10]. Following a consultation in 2016 on whether to reduce the availability of sugar-sweetened beverages (SSBs) on NHS premises, NHS England proposed a ban on SSBs should a voluntary reduction scheme not be effective at reducing the proportion of SSB sales to 10% or less by July 2018. Participating NHS Trusts met this threshold, so a national ban has not been established at this time. This requirement of 10% or fewer sales from SSBs now forms part of the Commissioning for Quality and Innovation specification – quality improvement goals that NHS Trusts can sign up to, with financial incentives – alongside the requirement than 80% of available confectionery and sweets do not exceed 250 kcal [11].

Investigating the effectiveness of altering the availability of food and drink options to encourage healthier purchasing within hospital settings is therefore valuable and timely. The current study investigated the impact of altering the absolute-and-relative availability of healthier and less healthy foods and cold beverages in vending machines in one NHS hospital that was part of a larger Trust. While in the daytime shops and cafes are open in hospitals, at night, vending machines are often the only option available from which staff, visitors or patients can purchase food. This study provides an initial investigation of the effect of altering the availability – both the overall number of options and the proportion of healthier to less healthy items simultaneously – in one setting in which there is desire for change, but where the potential impact has yet to be established. The aims were to estimate:

1.
  - The impact of decreasing the number of vending machine slots containing less healthy foods and drinks (and therefore the proportion of less healthy options as well) on the total energy purchased (kcal) per vending machine per week
  - The impact of increasing the number of vending machine slots containing healthier foods and drinks (and therefore the proportion of healthier options as well) on (i) the total energy purchased (kcal) per vending machine per week
2. Any difference in the impact of altering the number of vending machine slots containing healthier foods and drinks vs. the impact of altering the number of vending machine slots containing less healthy foods and drinks, on the total energy purchased (kcal) per vending machine per week
3. Any difference in the total number of items purchased per vending machine per week as a result of changing the number of slots containing (a) healthier or (b) less healthy foods and drinks

## Methods

### Design

An adapted multiple treatment reversal design was used in which all standard vending machines dispensing snack foods and/or cold beverages changed the number of slots containing (i) less healthy items and (ii) healthier items over seven 4-week periods between September 2017 and March 2018 (see Table 1). Cold drinks and snacks were treated separately given that at baseline the range of cold drinks offered had a mean of 56% (range 14–70%) for healthier products, while snacks had a mean of 25% (range 17–46%) healthier products. Combination machines (i.e. machines offering both snacks and cold drinks) were used in both cold drink and snack analyses. The study was registered at ClinicalTrials.gov (NCT03252158) and pre-registered on the Open Science Framework (https://osf.io/w5v2n/). Changes that needed to be made following the study registration are listed in the Appendix.

**Table 1:** Intervention schedule

	Example intervention periods	
	*Cold drinks*	*Snacks*
*Period 1: Standardised*	75% healthier; 25% less healthy; 0% gaps	25% healthier; 75% less healthy; 0% gaps
Period 2: Decrease A*	75% healthier; 5% less healthy; 20% gaps	25% healthier; 55% less healthy; 20% gaps
Period 3: Increase B*	95% healthier; 5% less healthy; 0% gaps	45% healthier; 55% less healthy; 0% gaps
*Period 4: Standardised*	75% healthier; 25% less healthy; 0% gaps	25% healthier; 75% less healthy; 0% gaps
Period 5: Decrease B*	55% healthier; 25% less healthy; 20% gaps	5% healthier; 75% less healthy; 20% gaps
Period 6: Increase A*	55% healthier; 45% less healthy; 0% gaps	5% healthier; 95% less healthy; 0% gaps
*Period 7: Standardised*	75% healthier; 25% less healthy; 0% gaps	25% healthier; 75% less healthy; 0% gaps

N.B. All study periods lasted 4 weeks.

* The example proportions show the intervention schedule for machines assigned to increasing healthier items first; for half the machines the proportions shown for periods 2 and 3 were switched with those for periods 5 and 6

Vending machines were randomised to one of two sequences for the seven study periods: ABCADEA or ADEABCA. In Condition A (study periods 1, 4 and 7), the proportions of healthier products were standardised across all vending machines so that 75% of all drinks were healthier and 25% of snacks. (The higher standardised proportion for drinks relative to baseline was chosen so that the proportions of healthier to less healthy would mirror those for snacks, but in reverse.) In Condition B, 20% of vending machine slots were emptied by removing less-healthy products. In Condition C, the empty slots created in Condition B were filled with healthier products. Conditions D and E were operationalised in the same way as B and C, except healthier products were removed from 20% of the vending machine slots in D, and then less-healthy products added in E.

In this design, the overall number of options (i.e. slots containing items) was altered during the 4-week periods when Conditions B and D were implemented – options were decreased by 20% when gaps were left in machines, compared to the other conditions. As such, this study examines the impact of altering absolute-and-relative availability [4] – i.e. both the overall number of options and the proportion of healthier to less healthy items vary simultaneously. The design separates out altering the number of slots containing healthier options and altering the number of slots containing less healthy options, allowing us to conduct a novel exploration of the different components underlying increasing the proportion of healthier items in vending machines (i.e. [1] increasing the number of healthier options and [2] decreasing the number of less healthy options), as well as enabling us to compare the impact of altering healthier vs. less healthy options.

### Sample

The sample comprised 10 vending machines serving a standard range of snack foods (*n* = 1), cold beverages (*n* = 3) or both (*n* = 6). As such, nine machines were eligible for the cold drinks part of the study, and seven for the snacks element. As this study used an opportunistic sample of available vending machines, a sample size calculation was not performed.

For those machines offering both snacks and cold drinks, the mean proportion of slots containing cold drinks was 38.9% (range: 36.8–42.9%). Machines branded as offering a healthier vending alternative were also present on site, but were not intervened upon, given they already had a higher proportion of healthier options than the standard range machines and were explicitly marketed as healthier alternatives, albeit without adhering to a particular definition of healthier options.

### Setting

The setting comprised vending machines serving a standard range of snack foods and/or cold beverages – i.e. not a healthier vending alternative – in the John Radcliffe Hospital, Oxford, England. Eight of the machines were accessible to staff, patients and visitors and two were accessible only to staff.

These machines were split between nine different locations in the hospital including outpatient clinic and A&E waiting rooms, and outside operating theatres (for staff only). Two machines were located together, one drink and one snack machine. These were allocated to the same intervention order. In seven of the nine locations, one or more of the vending machines branded as offering healthier options were also present (in total 14 vending machines branded as offering healthier alternatives were available at the hospital, across ten locations).

### Intervention

**Fig. 1:**
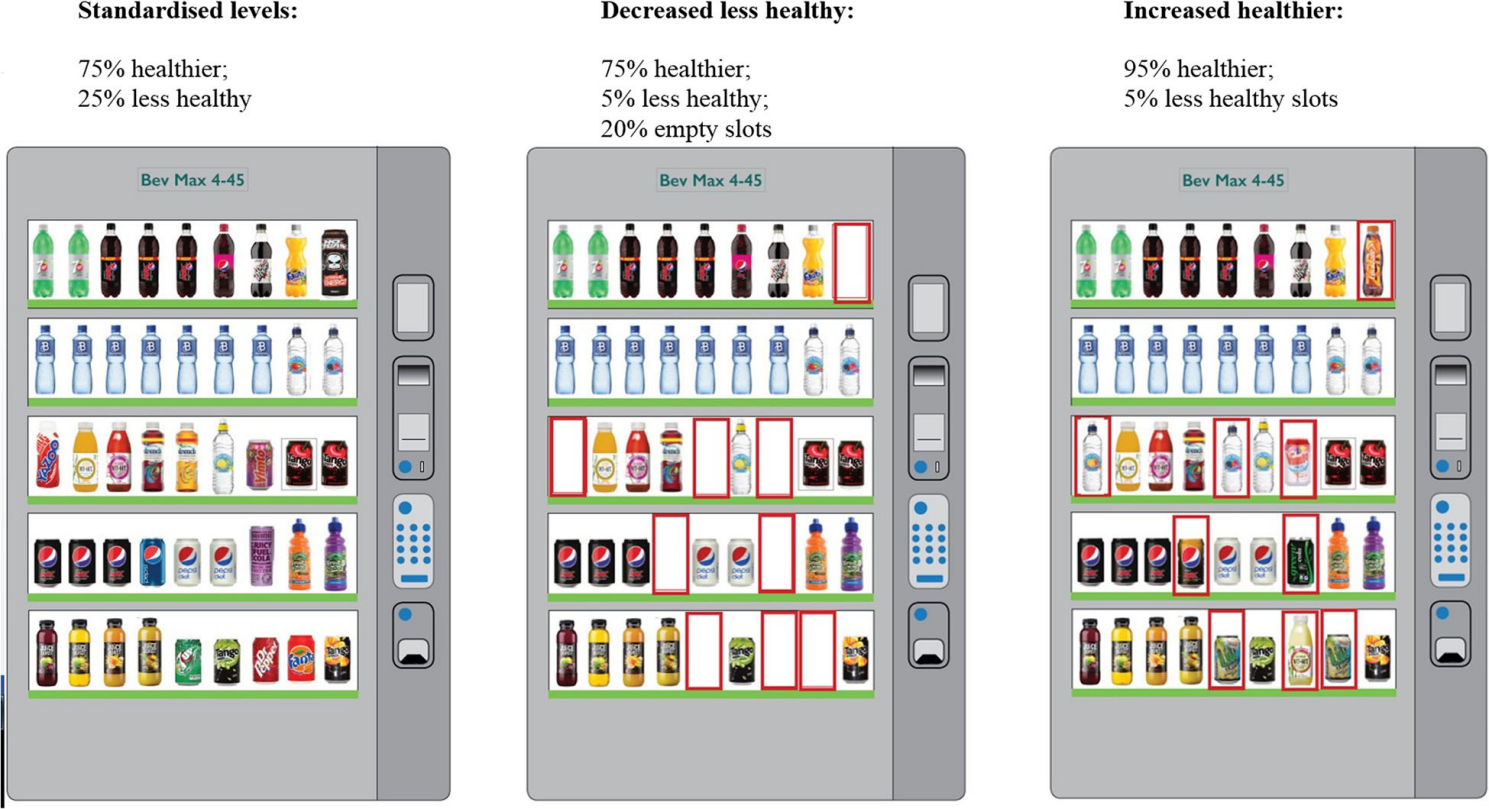
Example of changes made to one vending machine in the first three study periods

The intervention comprised altering the number of slots containing (a) healthier and (b) less healthy food or cold beverage options in hospital vending machines (see Table 1). Both reductions and increases corresponded to altering 20% of the available slots in each machine. See Fig. 1 for an example of the changes made to one vending machine in the first three study periods.

#### Healthier vs. less healthy foods and beverages

We applied the following criteria to define healthier and less healthy snacks and drinks:

- Snacks: Any snack food that contained more than 150 kcal (per package) was considered less healthy. This is based on the Change4Life guidance (http://www.nhs.uk/Change4Life/Pages/calories.aspx), which suggests a 400 kcal daily allowance for snacks and drinks. The 150 kcal threshold allows for 2 snacks a day, plus 100 kcal for drinks. While calories do not represent the whole picture with regard to healthiness, reducing the calorie intake from snacks, which are often regarded as ‘extras’ within the diet, has been one recommendation for healthier eating (e.g. Change4Life: http://www.nhs.uk/Change4Life/Pages/healthy-snacks.aspx). Exceptions were items that consist of fruit, nuts and seeds without added sugar or salt, which were classed as healthier. [No items qualified for this exception].
- Drinks: Any beverage containing 2.5 g or more of sugar per 100 ml was classed as less healthy (reflecting the threshold drink sugar content should fall below to allow a green traffic light label). The exception to this rule was 100% fruit juice [14/227 slots for drinks contained fruit juice; none were targeted in the intervention].

#### Altering the product range

When determining the items to be removed when decreasing options, the following criteria were used (in order of priority):

1. Replace slots containing duplicates of an exact product (e.g. if cans of Coca-Cola Zero were in three slots and cans of Fanta Zero Orange in two slots, a slot containing Coca-Cola Zero was replaced first).
2. Replace slots containing different versions or flavours of products from the same product range (e.g. if flavours of Yazoo are in three slots and flavours of Drench in two slots, a slot containing Yazoo was replaced first).
3. Products with the highest energy content (snacks) or sugar content per 100 ml (drinks) were then prioritised for replacement.

The majority of new products introduced into the machines did not duplicate existing products (177/180).

- For snacks, savoury replacements were found to replace savoury snacks, and sweet replacements for sweet snacks.
- For drinks, bottles and cans were matched in terms of their replacements. Where possible, flavours were matched – e.g. if a can of Lilt Zero was not already in the machine, then this would be used to replace a can of Lilt.

The number of products (e.g. Fanta, Fanta Zero) was kept constant as far as possible between the study conditions in which new items were introduced. Items were priced according to the vending machine supplier’s usual pricing – e.g. £1 for a 330 ml can. No additional labelling or signs indicating a change in available items were used.

### Procedure

Planograms were drawn up showing the product layout for each vending machine in each period, so that those stocking the machines could do so in accordance with these plans (see Additional file 1 for examples of planograms). Vending machines were filled at least weekly by the vending machine operator (more frequently for highly used machines), to match the planogram supplied for that machine and that period.

Data on the items placed in each machine at each restocking was recorded by the vending machine operator.

### Fidelity to protocol checks

Following each 4-weekly changeover, the vending machine operator took photos of each machine to send to the research team as a fidelity check. In addition, one day in the third week of each study period, one of the research team visited the site and took photos of each machine to ensure that each machine continued to be filled correctly.

### Analysis

The impact of the availability intervention was assessed in separate linear mixed models for cold drinks and snacks (run in Stata 15.1), examining the impact on total energy purchased (kcal) from drinks or snacks per week, with random effects for vending machine. Combination vending machines contributed to both analyses, with separate energy totals calculated from the drinks and snacks sold in each machine. Observations represented one week’s sales – as estimated from machine restocking – given all machines were restocked at least weekly. A *p*-value of < 0.0125 (two-tailed) was used to infer there was a statistically significant effect, using a Bonferroni adjustment to account for the four predictor variables being tested (*p* = 0.05/4) in analyses.

Main outcome: Energy purchased (kcal) per intervention per vending machine per week.

Secondary outcome: Number of items sold per week from intervention vending machines.

Predictor variables: A set of dummy variables characterising the changes made from the

standardised numbers of slots for healthier and less healthy items (for cold drinks, 75% healthier and 25% less healthy; for snacks, 25% healthier and 75% less healthy), namely:

1. slots containing less healthy items decreased from standard levels;
2. slots containing healthier items increased from standard levels;
3. slots containing less healthy items increased from standard levels;
4. slots containing healthier items decreased from standard levels.

Control variables included the number of items sold (for the main outcome), and dummy variables indicating study period (to allow for any seasonal trends in purchasing) and week within that period. The mean price of (i) healthier and (ii) less healthy items, and the number of slots filled in the machine were included as covariates.

Sensitivity analyses were run excluding outliers because very low numbers of restocked items were recorded for some machines for particular weeks, for example over the Christmas period for machines in some outpatient clinic settings. Given that this period affected machines differently depending on their location in the hospital (e.g. A&E sales were unaffected), it was not possible to control for these seasonal effects using dummy variables.

In addition, exploratory analyses compared the effects of increasing healthier (or less healthy) items on the proportion of purchased items classed as healthier.

### Missing data

- For the first week of each new study period, new products were loaded into each machine and some products were removed. However, the data recorded by the vending machine operator only listed the products loaded, and it was not possible to obtain counts of the number of items removed during these weeks. As such, the data for changeover weeks were not included in analyses, i.e. three weeks of data were analysed for each study period.
- On two occasions, no products were loaded in a vending machine for a particular week. Discussions with the vending machine supplier suggested it was likely that some products were sold but not in sufficient quantities to justify machine restocking. These values were therefore treated as missing in analyses, given they were unlikely to reflect true zeroes.
- In addition, one machine was out-of-order for the final study period.

## Results

### Baseline characteristics

Table 2 shows the baseline characteristics of the study vending machines. The mean energy per healthier snack product was 121 kcal, compared to 249 kcal for less healthy products. For drinks, the mean energy per healthier product was 18 kcal and per less healthy product was 121 kcal.

**Table 2:** Baseline characteristics of vending machines offering snacks and drinks

		Mean (SD)	
		Snacks	Drinks
Number of slots per machine		33 (9)	31 (12)
Number of unique products per machine		29 (6)	19 (6)
Energy (kcal) per slot	Healthier	121 (11)	18 (26)
	Less healthy	249 (74)	121 (53)
	Total	217 (85)	62 (65)
Number of healthier items sold per machine per week		43 (58)	57 (83)
Number of less healthy items sold per machine per week		136 (156)	47 (59)
Energy (kcal) per healthier product sold		120 (13)	11 (12)
Energy (kcal) per less healthy product sold		248 (69)	122 (49)

### Fidelity to protocol

Checks suggested fidelity to protocol was reasonable, with the wrong type of product (healthier or less healthy) being loaded or products not being loaded when requested in 7.6% (45/589) of changes required by the protocol (with many of these – 19 – occurring in study period 1). A small number of products (5 products, affecting 4.9% [29/589] of changes) had to be substituted for appropriate alternatives – i.e. matched in terms of being healthier or less healthy – part way through the study due to supply issues. Analyses were conducted on an intention-to-treat basis, with the restocking information for these products incorporated into the outcome measure.

### Impact on sales

**Fig. 2:**
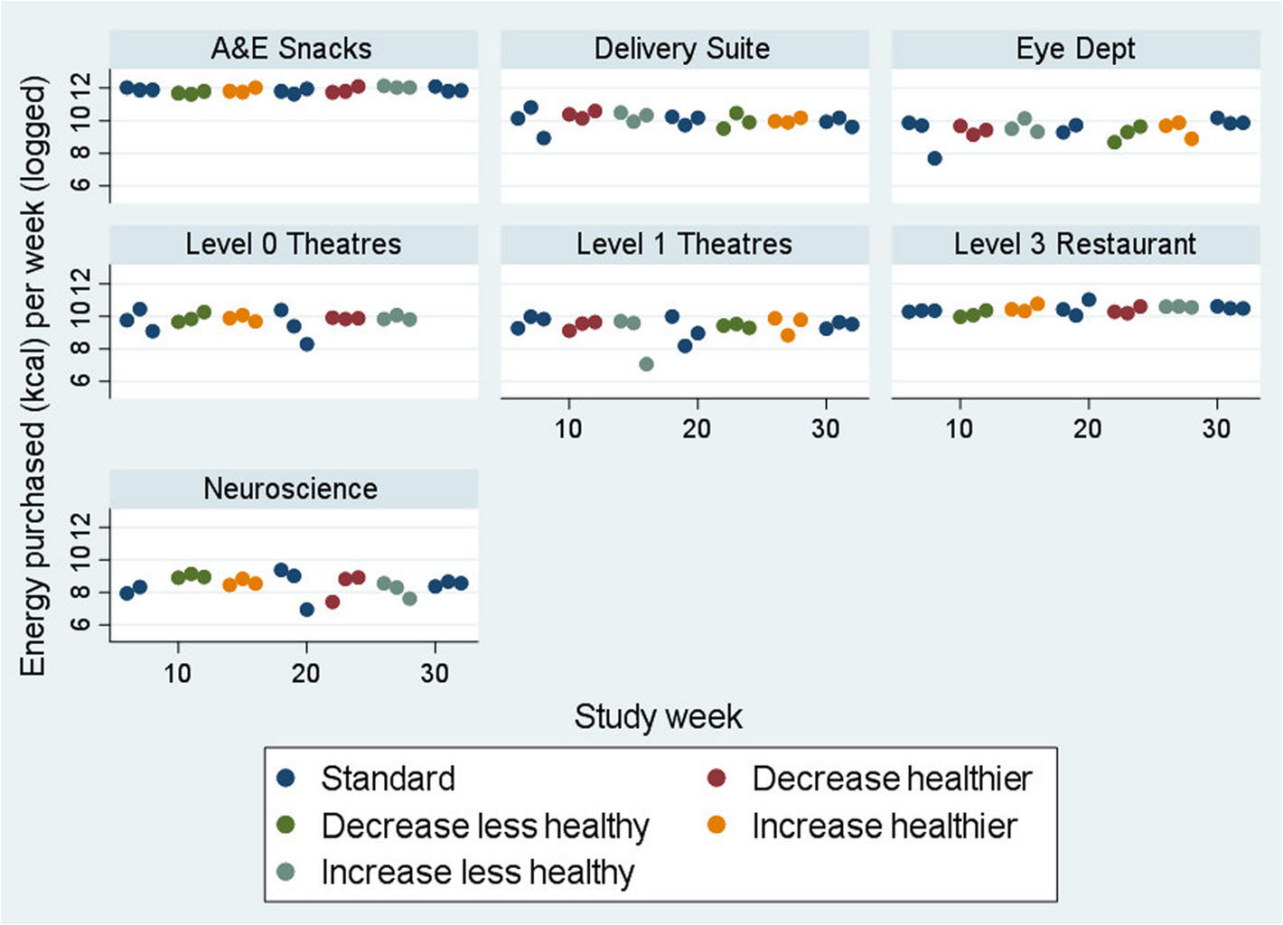
Energy purchased from snacks (kcal, logged) per week for snack machines. N.B. Increase healthier: Increase healthier in addition to decreased less healthy slots; Increase less healthy: Increase less healthy in addition to decreased healthier slots.

**Fig. 3:**
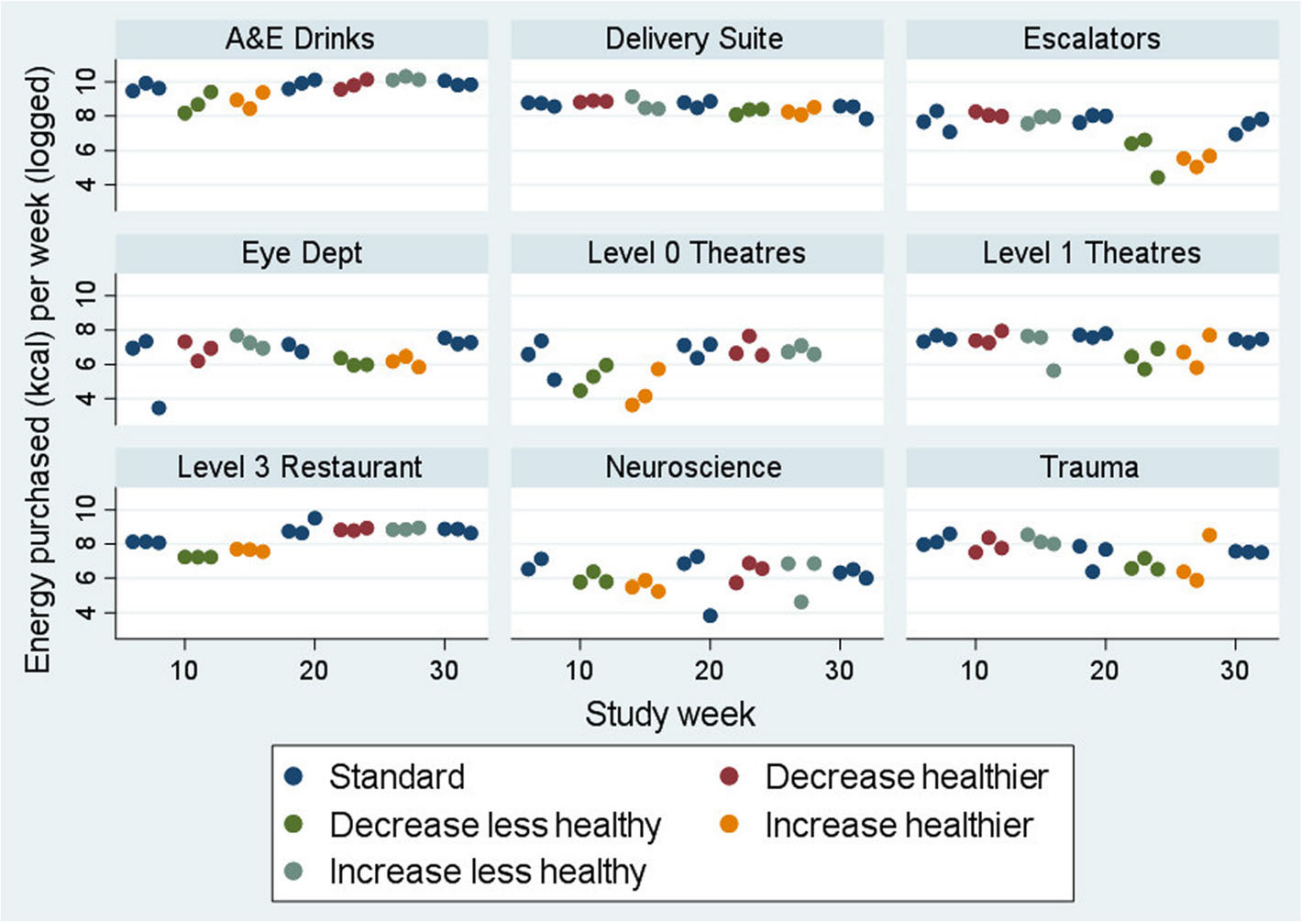
Energy purchased from drinks (kcal, logged) per week for cold drink machines. N.B. Increase healthier: Increase healthier in addition to decreased less healthy slots; Increase less healthy: Increase less healthy in addition to decreased healthier slots.

Figures 2 and 3 show the energy purchased per week for snack and drink machines, respectively. Due to wide variation by machine in mean sales (estimated from machine restocking), data are presented on the log scale.

Table 3 shows the mixed effect regression results for energy purchased for both snack and drink machines. Given the analysis was conducted with the outcome expressed on the log scale, regression coefficients have been exponentiated to represent the percentage change from standardised levels in energy (kcal) purchased in each availability condition.

**Table 3:** Mixed-effects regression results expressed as percentage change in energy purchased (kcal)

			Mean kcal per week per machine* (s.d.)	Percentage energy (kcal) change (95% CIs)	p-value (Kenward-Roger)
Snacks	Standard		37,813 (48,082)	*Ref*	*Ref*
	Decrease Less healthy		31,921 (38,622)	−17.2% (− 47.4, 30.5)	0.407
		& Increase Healthier	36,931 (46,198)	47.9% (−7.9, 137.4)	0.103
	Decrease Healthier		37,511 (47,705)	−19.1% (−48.5, 27.1)	0.350
		& Increase Less healthy	41,955 (56,127)	37.8% (−14.2, 121.3)	0.180
Drinks	Standard		4558 (5785)	*Ref*	*Ref*
	Decrease Less healthy		1750 (2592)	−52.6% (−69.3, −26.9)	0.001
		& Increase Healthier	2042 (2795)	−17.1% (−48.9, 34.6)	0.446
	Decrease Healthier		4900 (5863)	21.0% (−23.8, 92.1)	0.415
		& Increase Less healthy	5675 (7979)	3.1% (−37.4, 69.8)	0.903

* Unadjusted means; Means for increases represent periods when healthier options were increased while less healthy options remained decreased or periods when less healthy options were increased while healthier options remained decreased

Relating to Aim 1, energy purchased from snacks was not significantly altered by decreasing the number of slots containing less healthy options (Aim 1a; − 17.2%; 95% CI: − 47.4, 30.5), or increasing the number of slots containing healthier options (Aim 1b; 47.9%; 95% CI:-7.9, 137.4). Nor was energy purchased from snacks significantly altered by the reverse conditions, which similarly showed non-significant decreases in energy purchased when slots were reduced (Healthier: − 19.1%; 95% CI: − 48.5, 27.1), and non-significant increases when new products were added (Less healthy: 37.8%; 95% CI: − 14.2, 121.3).

Energy purchased from drinks was lower when the number of slots containing less healthy drinks was decreased, compared to standardised levels (− 52.6%; 95% CI: − 69.3, − 26.9) (Aim 1a). A non-significant decrease in energy purchased from drinks was seen when healthier drinks were then added into the emptied slots (− 17.1%; 95% CI: − 48.9, 34.6) (Aim 1b). Both decreasing the number of slots for healthier drinks, and subsequently filling those slots with less healthy drink options, led to non-significant increases in energy purchased (Decrease Healthier: 21.0%; 95% CI: − 23.8, 92.1; Increase Less healthy: 3.1%; 95%CI: − 37.4, 69.8).

### Sensitivity analyses

Given the large variation in sales week-by-week, analyses were re-run removing any outliers (defined as having standardised residuals greater than 3 or less than − 3). These analyses (removing two observations for snacks and two for drinks where very low sales were recorded) did not change the pattern of results.

### Comparison of targeting healthier and less healthy products

In order to address Aim 2, the sizes of the effects for altering healthier vs. less healthy foods or drinks obtained in Table 3 were compared. Stata’s ‘lincom’ command was used to combine regression coefficients in order to contrast healthier vs. less healthy changes for (a) Increased availability and (b) Decreased availability. These analyses aim to explore the extent to which altering (increasing/decreasing) less healthy items change energy purchased to a greater extent than the equivalent change (increase/decrease) to healthier items. The computed coefficients (and 95%CIs) showed no significant differences:

- Snacks: Increased availability: − 0.07 (− 0.40, 0.26), *p* = 0.67;
  Decreased availability − 0.02 (− 0.31, 0.26), *p* = 0.87;
- Drinks: Increased availability: − 0.16 (− 0.98, 0.67), *p* = 0.71;
  Decreased availability: 0.56 (− 0.26, 1.37), *p* = 0.18.

### Secondary analysis

Table 4 presents the results of analyses assessing the impact of the study conditions on the number of items restocked each week (Aim 3). None of the percentage changes reached the threshold of *p* < 0.0125. Moreover, in sensitivity analyses removing outliers (defined as above), no *p*-value fell below *p* < 0.05.

**Table 4:** Mixed-effects regression results expressed as percentage change in the number of items purchased

			Mean number of items sold per week per machine* (s.d.)	Percentage change (95% CIs)	p-value (Kenward-Roger)
Snacks	Standard		167 (209)	*Ref*	*Ref*
	Decrease Less healthy		155 (191)	77.8% (3.6, 205.2)	0.037
		& Increase Healthier	182 (226)	−27.4% (− 58.9, 28.1)	0.262
	Decrease Healthier		149 (185)	64.1% (−3.6, 179.5)	0.067
		& Increase Less healthy	171 (224)	− 39.5% (− 65.5, 6.2)	0.078
Drinks	Standard		99 (135)	*Ref*	*Ref*
	Decrease Less healthy		74 (96)	12.5% (−21.0, 60.2)	0.509
		& Increase Healthier	79 (97)	13.4% (−41.1, 27.3)	0.461
	Decrease Healthier		94 (123)	38.0% (−5.3, 101.1)	0.093
		& Increase Less healthy	103 (155)	−29.2% (− 52.6, 5.7)	0.091

* Unadjusted means; Means for increases represent periods when healthier options were increased while less healthy options remained decreased or periods when less healthy options were increased while healthier options remained decreased

### Exploratory analyses

**Fig. 4:**
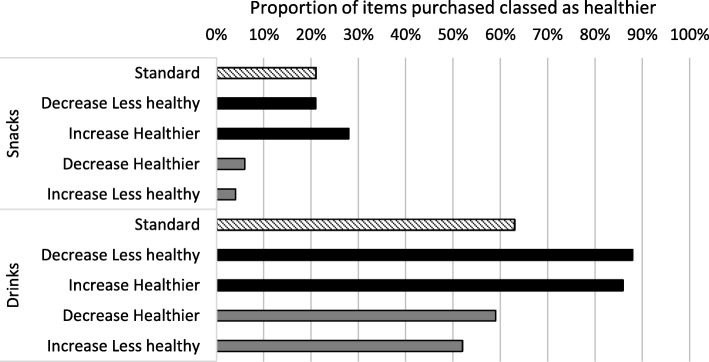
Proportion of items purchased classed as healthier, by study condition, for snacks and drinks. N.B. Increase healthier: Increase healthier in addition to decreased less healthy slots; Increase less healthy: Increase less healthy in addition to decreased healthier slots.

Figure 4 shows the impact of the study condition on the proportion of purchased items classed as healthier.

Further exploratory analyses examining the impact of altering availability on the proportion of purchased items classed as healthier reflected the results for energy purchased (see Additional file 1), whereby for drinks, decreasing the number of slots available for less healthy options resulted in a 24-percentage point increase (95%CI: 0.17, 0.32) in the proportion of healthier drinks sold, with no other coefficients reaching statistical significance.

## Discussion

This study presents a robust evaluation of altering the absolute-and-relative availability of healthier vs. less healthy items in a field study of vending machines in an English hospital. The results suggest that decreasing the number of slots available for less healthy drink options – from 25 to 5% of slots, while maintaining 75% slots containing healthier options – reduced energy (kcal) purchased by 53% (95%CI: 27, 69%). There was no evidence that overall sales were reduced – based on analyses examining the impact of these changes on the number of items restocked – when decreasing the number of slots available for less healthy drink options. As such, this reduction in energy purchased seems likely to have been driven by a change in the types of item purchased rather than reduced sales, supported by exploratory analyses suggesting a 24-percentage point increase in the proportion of healthier items sold under this study condition. In comparison, the recent study by Public Health England [6] suggested a 36% reduction in energy purchased when the ratio of slots available for SSBs and non-SSBs was altered from around 50:50 to 20:80 (SSB:non-SSB), albeit with SSBs being placed in prominent positions. The current study provides a more robust evaluation of this type of intervention, and suggests that making further reductions beyond the 20% of slots for less healthy drinks would continue to impact on purchasing behaviour.

No statistically significant differences in energy purchased were seen for increasing the number of slots available for healthier drinks on top of decreasing less healthy slots. This inconclusive finding might reflect limited power to detect other than a large effect due to the small opportunistic sample available in the current study. If replicated in larger studies, however, this could suggest that customers may be more sensitive to changes to less healthy over healthier drink options (findings directly comparing the sizes of the effects for altering healthier over less healthy drinks were also inconclusive in the current study). This would reflect recent findings from an online selection task showing greater sensitivity to the presence of less healthy snack foods than to healthier snack foods [12]. This is also in keeping with observational data suggesting that the availability of less healthy foods but not fruit and vegetables is associated with body mass index (BMI) [13]. One possible explanation for this pattern of findings is that less healthy options are harder to resist – with evidence suggesting that response inhibition (which predicts obesity and food-related behaviour [14–17]) has a more limited impact on consumption of healthier foods [18–20]. The effects of response inhibition on dietary behaviour may also be stronger when the foods involved are more appealing [21–24], an effect in part due to repeated pairings of less healthy foods with positive unconditioned stimuli through marketing [25–27].

This study did not suggest any consistent pattern in results between snacks and drinks, with no statistically significant differences in energy purchased from snacks found as a result of altering availability in snack machines. Exploratory analyses suggested potentially larger effects for decreasing healthier rather than less healthy items for snacks (contrary to the results for drinks), with an 11-percentage point reduction in the proportion of healthier items sold for decreasing the number of slots available for healthier snack options (see Additional file 1). However, this did not reach statistical significance, perhaps due to limited power given the small sample size. This might be due to the different baseline proportions of healthier items (25% for snacks vs. 75% for drinks). Altering snacks and drinks might also result in different substitution patterns – for example, people may be more willing to swap to a diet version if their usual full-sugar drink option is unavailable, but be less likely to select a healthier snack option if their favoured snack is not available. This could tie in with findings from the recent Public Health England report [6], which also observed smaller reductions in energy for snacks compared to drinks (10% vs. 36%) when limiting the number of vending machine slots available for less healthy products. It should be noted that the changes made in the latter study were predominantly focused on replacing savoury snacks, which may have smaller energy differences per packet.

### Strengths and limitations

This study is the first to our knowledge to isolate the impact of altering the absolute-and-relative availability – i.e. simultaneously altering the overall number and proportion – of (a) healthier vs. (b) less healthy options in a field setting. The study examined both snacks and cold drinks, the standard proportions of healthier options being quite distinct for these two categories, allowing us to explore the effects of this intervention given these different baselines in availability. In addition, the study separated out the impact of the intervention on overall sales and types of items purchased – healthier and less healthy – to establish the manner in which implementing this intervention might impact on energy purchased.

It should be noted that our manipulation of the number of slots containing healthier and less healthy items would also have impacted on the range of products available. While this was minimised as far as possible when decreasing the number of slots assigned to healthier or less healthy items, this inevitably changed when the number of healthier or less healthy items was already small, necessitating the removal of some unique products. Similarly, when increasing the presence of healthier or less healthy items, new items were introduced, thus the range of products available also increased. In addition, the presence of new items in the increased availability conditions might lead to increased purchases of these products due to a novelty effect – highlighting the importance of further studies examining the persistence of effects over time.

Other limitations of this study include the relatively small number of vending machines in our sample. As a result, the study had limited power, so null results should be interpreted with caution. We were also unable to account for other possible compensatory purchasing due to the availability of other food vendors on site. Data represent the items loaded into machines each week by the vending machine operator, rather than sales per se – for example, if very low sales of a particular item had taken place over a week, the operator may not have fully restocked that product. The operator also did not record the number of items removed during weeks when the stock was changed due to the study interventions, meaning that data for those weeks could not be used in analyses, reducing study power.

### Implications for research and policy

The study results suggested that reducing the proportion of less healthy cold drink options available halved the energy purchased from drinks in the hospital vending machines, without evidence of a reduction in overall sales. Findings were inconclusive for snacks. This study offers support for policies that limit the proportion of less healthy drink options available in hospitals [11] and other public and private sector settings. Given that there was no evidence that the intervention impacted on overall sales as estimated from machine restocking, it may be possible to implement this intervention without loss of revenue to the vending machine operator.

In the current study, we defined healthier options in terms of lower energy for snacks. Further studies could examine the impact of looking at different ways of defining healthier options including considering sugar/fat/salt content. Further exploring the impact of baseline proportions of healthier items and the degree of change that might be required to see an impact on energy purchased would help to establish the best ways of implementing this promising intervention across different settings.

## Conclusion

Reducing the proportion of less healthy cold drink options available in vending machines from 25 to 5% of slots halved the energy purchased from drinks, with no evidence of a reduction in overall sales (as estimated from machine restocking) in this study, suggesting this is a promising strategy for encouraging healthier drink selections. Further research is needed to establish whether any effects might be smaller for snacks, or found with higher baseline proportions of healthier options.

### Supplementary information

**Additional file 1.** Example planograms and exploratory analyses examining the proportion of sales classed as healthier.

## Data Availability

The data are commerically sensitive, so are not available to share beyond the research team.

## Appendix

Appendix: Changes to published protocol.

Some changes were made following the trial registration (ClinicalTrials.gov: NCT03252158; Open Science Framework: https://osf.io/w5v2n/):

- Covariates:
  - Some machine-level covariates (presence of healthier-fare vending machines/other food outlets nearby; number of slots in machine) were listed in the original registration. Given that these were consistent over time, there was no need to include these as well as the random effects by machine in analyses.
  - The presence of gaps in machine was listed as a covariate in the study registration, but given this was closely linked to study period and the intervention dummy variables, it was too highly correlated to include. The number of slots filled in each machine was used instead.
  - Rather than calendar month, dummy variables indicating study period and week within period were used to specify time. Calendar month sometimes but not always mapped onto study period, making this hard to interpret, so using study period instead was preferred. Week within period was added to account for any novelty effects of changes.
- Secondary outcomes relating to (healthier-fare) vending machines: Analyses looking at how the intervention affected sales at healthier-fare vending machines were originally planned. However, due to these machines being stocked less frequently (often fortnightly), limited data were available. Moreover, given that the intervention varied by machine (e.g. some reducing healthier and some less healthy during the same period), it would not have been possible to assess the impact of a particular intervention on sales at these machines.
