# Supplementary material for: Altering the availability of healthier vs. less healthy items in UK hospital vending machines: a multiple treatment reversal design
Source: Int J Behav Nutr Phys Act. 2019 Nov 27;16:114. doi: 10.1186/s12966-019-0883-5 (PMC6882209; doi:10.1186/s12966-019-0883-5)
Supplement: Supplementary file 1 — Additional file 1. Example planograms and exploratory analyses examining the proportion of sales classed as healthier. [file 12966_2019_883_MOESM1_ESM.docx]

**Supplementary Materials**

***Example planograms:*** A&E Drinks Machine

1^st^ Study Period: 75% healthier; 25% less healthy slots


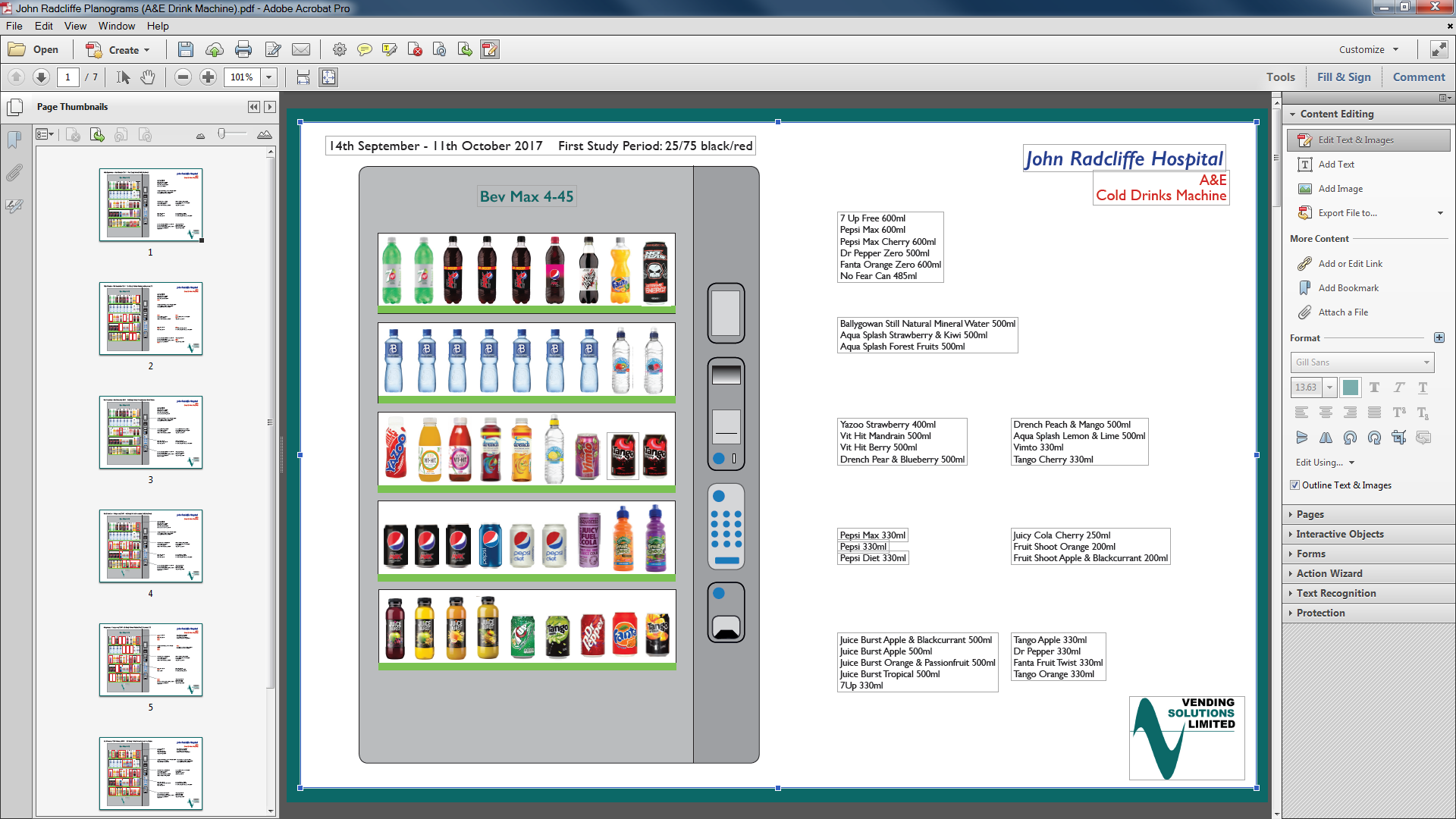


2^nd^ Study Period: 75% healthier; 5% less healthy; 20% empty slots


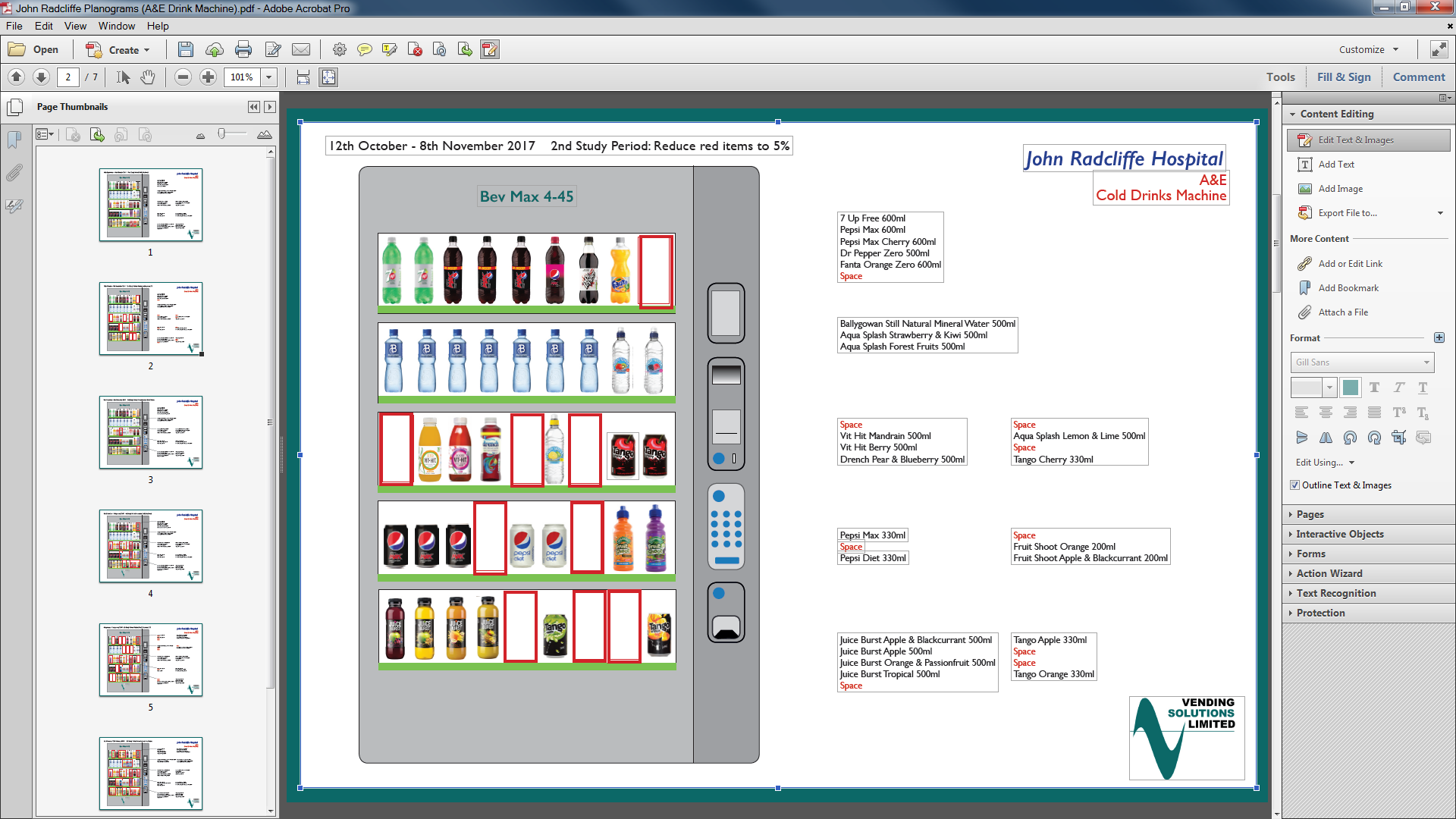


3^rd^ Study Period: 95% healthier; 5% less healthy slots


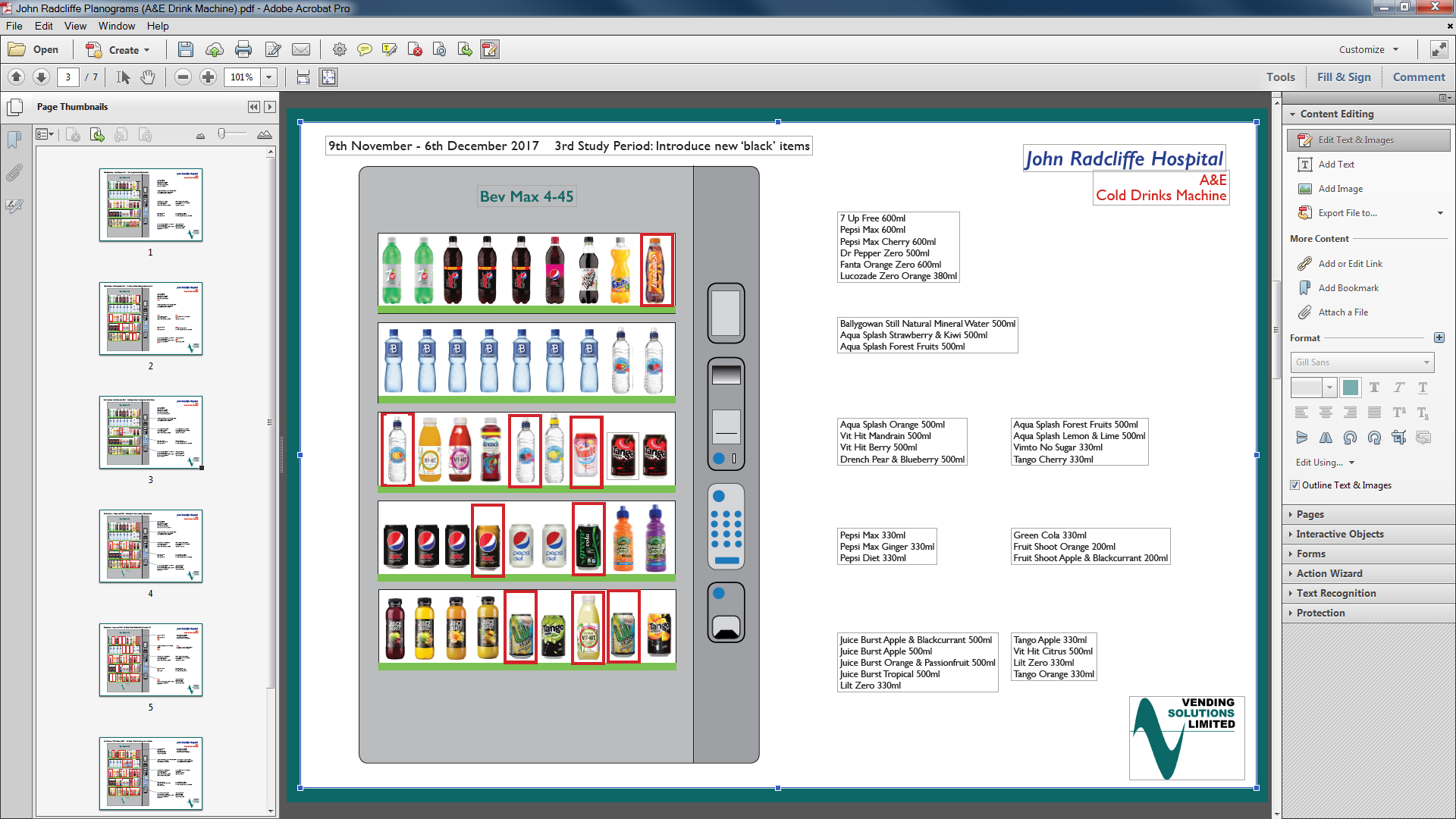


4^th^ Study Period: 75% healthier; 25% less healthy slots


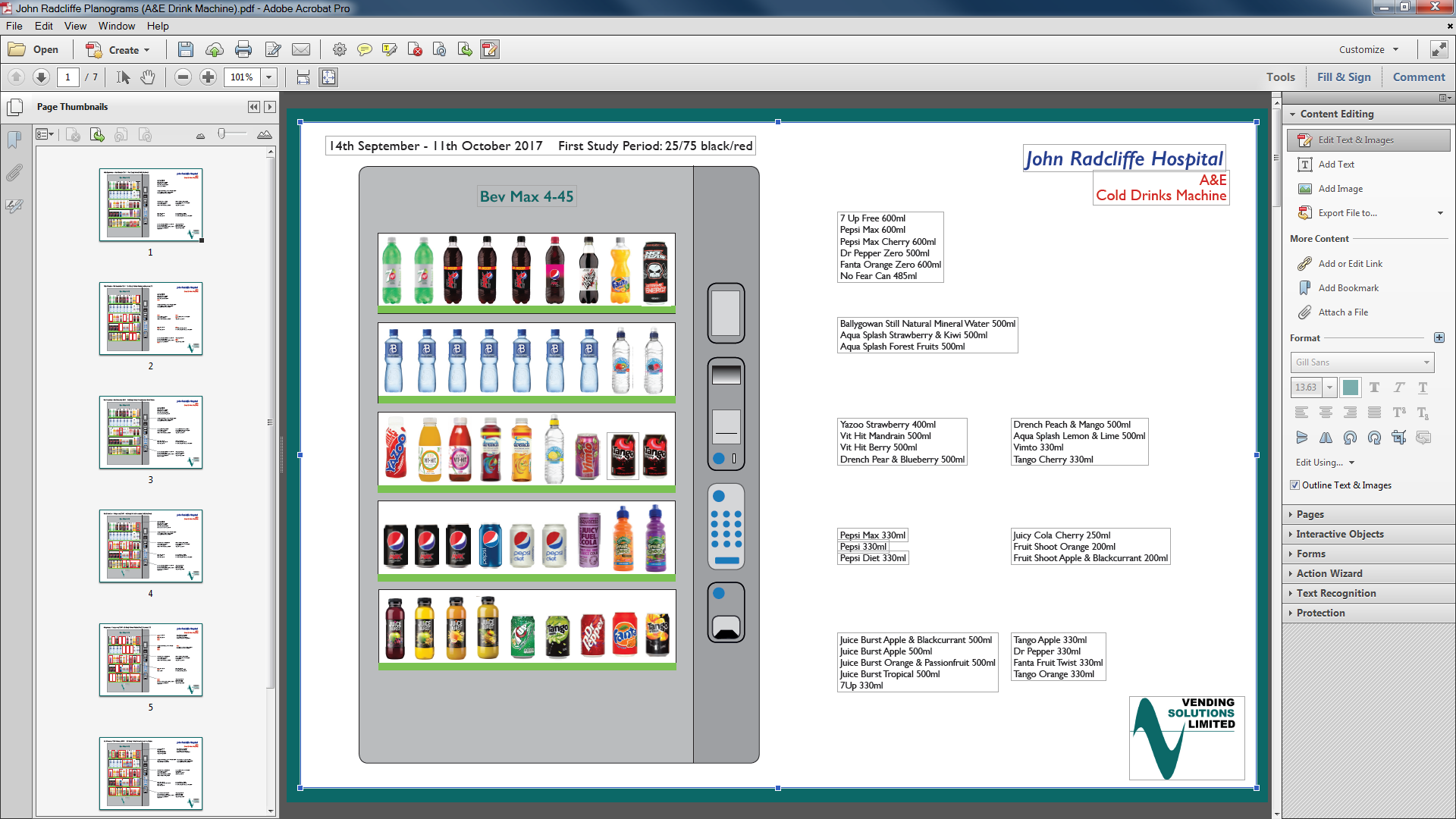


5^th^ Study Period: 55% healthier; 25% less healthy; 20% empty slots


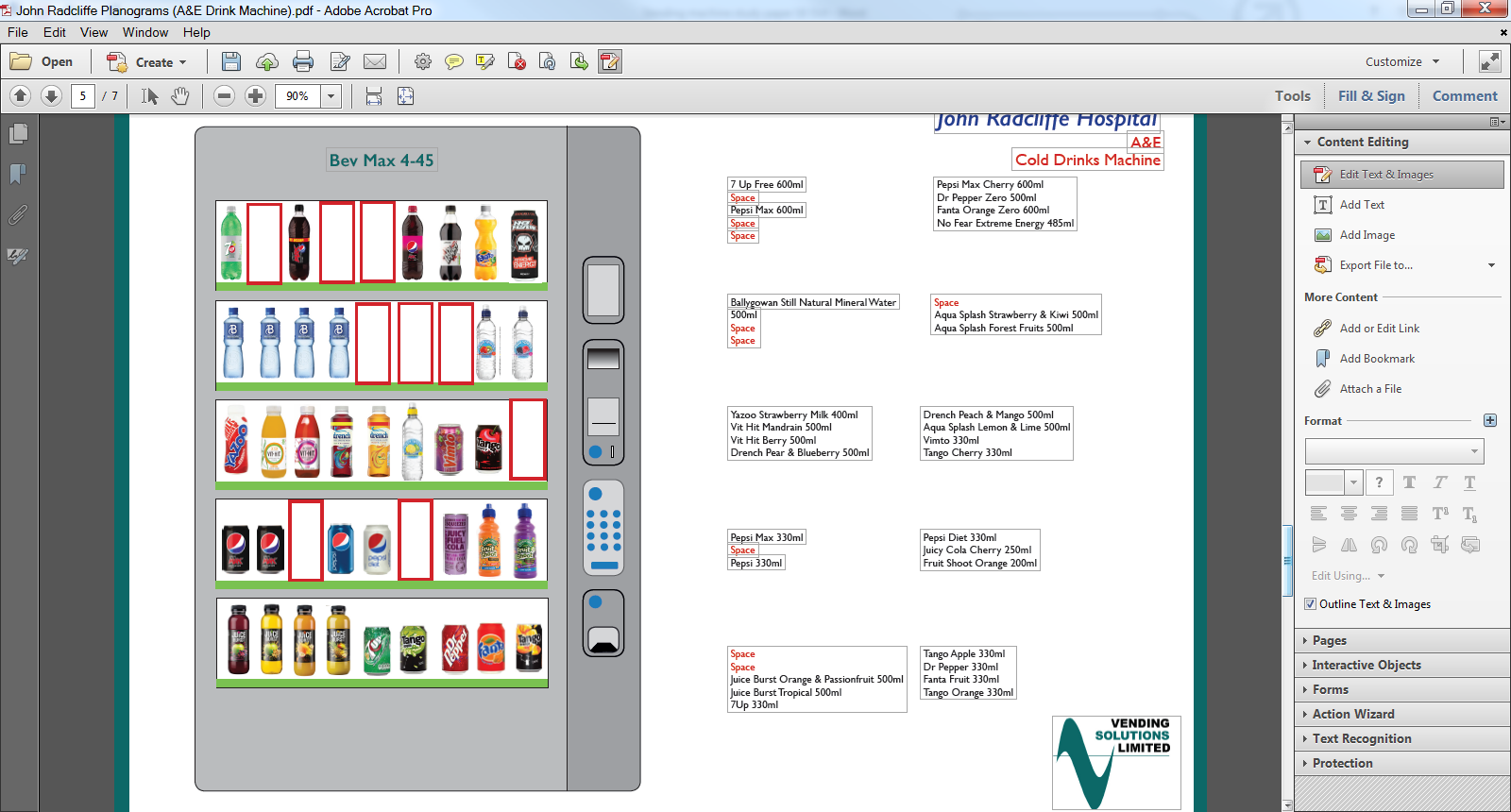


6^th^ Study Period: 55% healthier; 45% less healthy slots


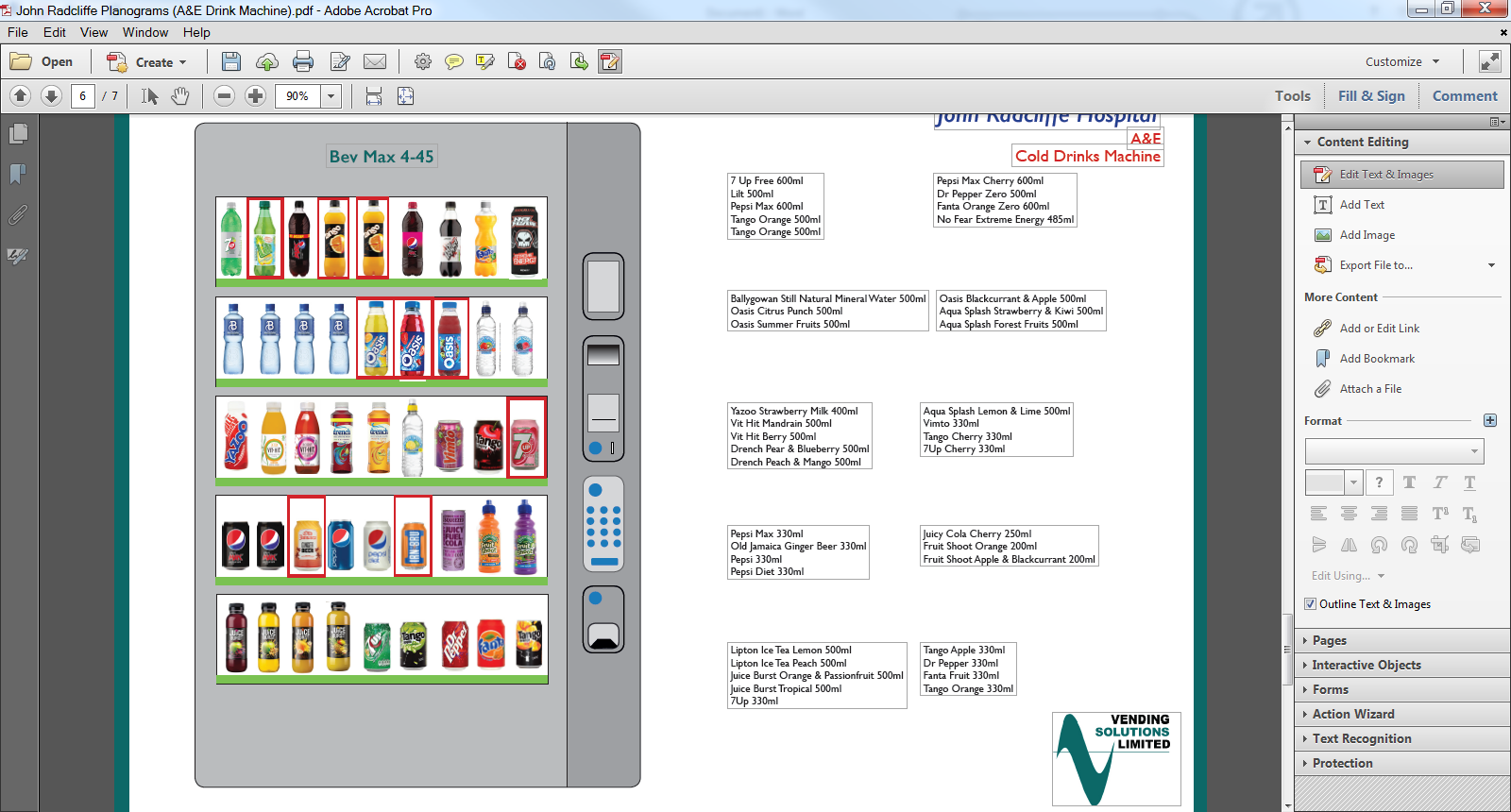


7^th^ Study Period: 75% healthier; 25% less healthy slots


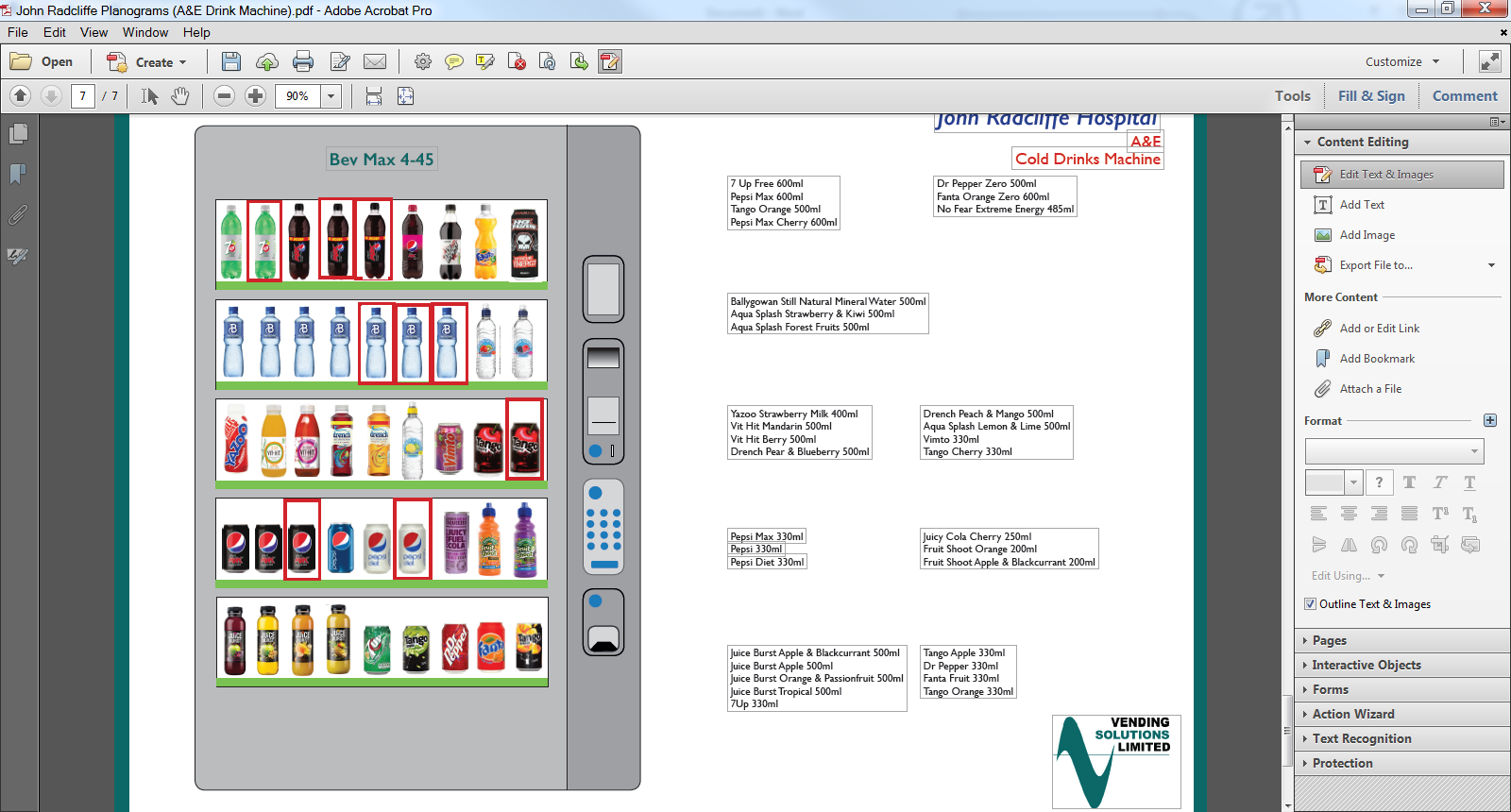


***Exploratory analyses:*** Taking proportion of sales classed as healthier as outcome

In order to explore the impact of the study condition on the proportion of purchased items classed as healthier, exploratory analyses were conducted (see Table S1).

**Table S1:** Mixed-effect Tobit regression results for proportion of purchased items classed as healthier

|  | | | **Mean proportion healthier items sold per week per machine* (s.d.)** | **Coefficient**  **(95% CIs)** | **p-value**** |
| --- | --- | --- | --- | --- | --- |
| Snacks | Standard | | 21%  (16) | *Ref* | *Ref* |
|  | Decrease Less healthy | | 21%  (11) | 0.06  (-0.05, 0.16) | 0.277 |
|  |  | & Increase Healthier | 28%  (12) | 0.07  (-0.05, 0.18) | 0.251 |
|  | Decrease Healthier | | 6%  (9) | -0.11  (-0.22, -0.01) | 0.036 |
|  |  | & Increase Less healthy | 4%  (7) | -0.04  (-0.16, 0.07) | 0.463 |
| Drinks | Standard | | 63%  (13) | *Ref* | *Ref* |
|  | Decrease Less healthy | | **88%**  **(9)** | **0.24**  **(0.17, 0.32)** | **0.000** |
|  |  | & Increase Healthier | 86%  (13) | 0.01  (-0.08, 0.10) | 0.838 |
|  | Decrease Healthier | | 59%  (13) | -0.04  (-0.12, 0.04) | 0.362 |
|  |  | & Increase Less healthy | 52%  (19) | -0.11  (-0.20, -0.02) | 0.018 |

* Means for increases represent periods when healthier options were increased while less healthy options simultaneously decreased or periods when less healthy options were increased while healthier options simultaneously decreased

** Unable to apply Kenwood-Roger adjustment to Tobit regression

These analyses reflect those for energy purchased, whereby for drinks, decreasing the number of slots available for less healthy options resulted in a 24-percentage point increase (95%CI: 0.17, 0.32) in the proportion of healthier items sold. However, it should be noted that the Kenwood-Roger adjustment for small sample size could not be applied to these Tobit regression, so these statistical significance levels have not been adjusted.
